# Access to innovative drugs for metastatic lung cancer treatment in a French nationwide cohort: the TERRITOIRE study

**DOI:** 10.1186/s12885-018-4958-5

**Published:** 2018-10-22

**Authors:** Arnaud Scherpereel, Isabelle Durand-Zaleski, François-Emery Cotté, Jérôme Fernandes, Didier Debieuvre, Cécile Blein, Anne-Françoise Gaudin, Charlène Tournier, Alexandre Vainchtock, Pierre Chauvin, Pierre-Jean Souquet, Virginie Westeel, Christos Chouaïd

**Affiliations:** 1 Pulmonary and Thoracic Oncology Department, CHU de Lille, Lille University, Lille, France; 2 URCEco Île-de-France, Hôtel-Dieu Hospital, Paris, France; 3 Department of Public Health, Henri-Mondor Hospital, Créteil, France; 4 Health Economics and Outcomes Research, Laboratoire Bristol-Myers Squibb, Rueil-Malmaison, France; 5 OC Santé, Montpellier, France; 6 Department of Chest Medicine, GHRMSA, Emile Muller Hospital, Mulhouse, France; 7 HEVA, Lyon, France; 8 Institut Pierre Louis d’Epidémiologie et de Santé Publique (IPLESP UMRS 1136), Department of Social Epidemiology, Sorbonne Universités, UPMC Université Paris 06, INSERM, Paris, France; 9 Department of Chest Medicine, Hospices Civils de Lyon, Centre Hospitalier Lyon Sud, Pierre-Bénite, France; 10 Department of Chest Medicine, Jean Minjoz University Hospital, Besançon, France; 11 Department of Chest Medicine, Créteil University Hospital, Créteil, France

**Keywords:** Metastatic lung cancer, Innovative treatments, France, Social deprivation

## Abstract

**Background:**

Territorial differences in the access to innovative anticancer drugs have been reported from many countries. The objectives of this study were to evaluate access to innovative treatments for metastatic lung cancer in France, and to assess whether socioeconomic indicators were predictors of access at the level of the municipality of residence.

**Methods:**

All incident cases of metastatic lung cancer hospitalised for a chemotherapy in public hospitals in 2011 were identified from the French National Hospital discharge database. Information on prescription of innovative drugs from an associated database (FICHCOMP) was crossed with the population density of the municipality and a social deprivation index based on national census data.

**Results:**

Overall, 21,974 incident cases of metastatic lung cancer were identified, all of whom were followed for 2 years. Of the 11,486 analysable patients receiving chemotherapy in the public sector, 6959 were treated with a FICHCOMP drug at least once, principally pemetrexed. In multivariate analysis, prescription of FICHCOMP drugs was less frequent in patients ≥66 years compared to those ≤55 years (odds ratio: 0.49 [0.44–0.55]), in men compared to women (0.86 [0.79–0.94]) and in patients with renal insufficiency (0.55 [0.41–0.73]) and other comorbidities. Prescription rates were also associated with social deprivation, being lowest in the most deprived municipalities compared to the most privileged municipalities (odds ratio: 0.82 [0.72–0.92]). No association was observed between the population density of the municipality and access to innovative drugs.

**Conclusion:**

Although access to innovative medication in France seems to be relatively equitable, social deprivation is associated with poorer access. The reasons for this need to be investigated and addressed**.**

## Background

The total health expenditure on cancer in the European Union in 2014 has been estimated at € 83.2 billion, an increase of 65% over the previous 20 years [1]. Of all tumours, lung cancer has the highest economic cost, amounting to 15% of the overall cancer costs followed by breast cancer (12%), colorectal cancer (10%) and prostate cancer (7%) [2]. Acquisition costs for anticancer drugs as a proportion of total treatment cost have increased from 12% in 1995 to 23% in 2014 [1]. The increase in the acquisition costs of anticancer drugs can in part be attributed to the introduction of innovative therapies (monoclonal antibodies, small molecule targeted therapies and more recently immunotherapies) which have permitted major gains in survival for certain cancers compared to previous generations of drugs. This has allowed such treatments to command premium prices from payers. However, an untoward consequence of the expense of these drugs was to restrict access to treatment, in particular in health systems where healthcare budgets are delegated to a local or hospital level, with marked territorial differences in access to treatment. This issue of ‘postcode prescribing’ became particularly problematic in the United Kingdom before measures were taken to improve equality of access to treatment [3, 4]. Recent studies from Canada [5–7] and Italy [8] have also demonstrated inequalities of access to drugs for cancer due to differences in access policies between regions within countries.

In France, the National Institute of Cancer estimated the direct medical cost of cancer to be € 11 billion in 2004 [9], rising to € 15 billion in 2014, of which anticancer drugs accounted for 22% [1]. In the disease-related group (DRG) payment scheme in French hospitals, the acquisition cost of most drugs is included in the unit cost of hospitalisation. Since the introduction of this scheme, a special mechanism was implemented to facilitate access to innovative expensive drugs administered during hospital stays. A list of such drugs (called *liste en sus*) was established, based on prespecified criteria for innovation, which are funded separately from the DRG-based payments [10]. The decision to use one of these drugs is taken on a patient-by-patient basis during a multidisciplinary care team (MCT) meeting in which potential risks and benefits of treatment are evaluated. This system allows hospitals to be fully reimbursed retrospectively, based on maximum reimbursement prices set by the Pricing Committee of the French Health Authority (*Comité Economique des Produits de Santé*, CEPS). Therapies included in this list are fully reimbursed up to reimbursement tariffs. Payment comes out of a specific national budget that protects hospitals from the costs of expensive new drugs. The goal is to ensure rapid diffusion of innovative therapies and to minimise the risk of ‘postcode prescribing’. The list of eligible treatments is reviewed annually and all drug use funded through this mechanism is documented in a specific database (FICHCOMP). At the time of the analysis, access to the FICHCOMP database was only available for public hospitals.

Since the introduction of the extra-DRG funding of innovative and expensive drugs, limited data is available regarding the performance of this system in minimising inequalities in access to anticancer drugs in the public domain. The objectives of the present study were to assess access to these treatments by patients with metastatic lung cancer using the French hospital discharge database and to assess whether socioeconomic indicators were predictors of access at the level of the municipality of residence.

## Methods

### Study design

The TERRITOIRE study was a historical cohort analysis of a medico-administrative database. Data was extracted from the French National Hospital discharge database (PMSI; *Programme de Médicalisation des Systèmes d’Information*) relating to all patients hospitalised in the public sector, crossed with geographically aggregated socioeconomic variables at the lowest local authority level, documented in the databases of the national census of the French national statistics office (INSEE) through individual patient postcodes.

### Hospital discharge database

The PMSI hospital discharge database covers all hospitalisations in the public and private sectors involving short-term stays in medical, surgical or obstetric facilities, representing more than 95% of all hospitalisations in France [11]. The reasons for hospitalisation are coded by International Classification of Diseases, 10th revision (ICD-10) codes [12], either as principal diagnoses (PD; the condition for which the patient was hospitalised), related diagnoses (RD; any underlying condition which may have been related to the PD) or as significantly-associated diagnoses (SAD; comorbidities which may affect the course or cost of hospitalisation). Sociodemographic data is limited to age at inclusion, gender and home address postcode. Patients can be tracked across multiple hospitalisations through a unique anonymous patient identifier, which is retained until the patient dies.

### Study population

The analysis included all patients with a documented ICD-10 code for any form of lung cancer (C34: Malignant neoplasm of bronchus and lung) as PD, RD or SAD for any public hospital stay in 2011 and, in order to restrict the sample to incident cases, without a previous hospitalisation with an ICD-10 code for lung cancer during the period 2006–2010. Metastatic disease was identified from three different sources, namely an ICD-10 code for metastatic disease, hospitalisation in palliative care as a first hospitalisation for lung cancer or administration of chemotherapy for metastatic disease.

### Data collection

At the first documented hospitalisation for metastatic lung cancer (index visit), the gender and age of each patient was documented, as well as the presence of significant chronic comorbidities (hypertension, diabetes mellitus, renal insufficiency and other chronic lung diseases). The choice of these comorbidities was justified by the fact that these are systematically taken into account during the MCT meeting at which the decision to use a FICHCOMP drug is made. The type of hospital at which the patient was diagnosed was identified. Data was collected for the two-year period following the index hospitalisation.

#### Chemotherapy

All chemotherapy sessions over the two-year follow-up period which involved treatments listed in the FICHCOMP database were identified. Only specific anticancer therapies which were commercially available during the observation period were considered. It should be noted that many patients with metastatic lung cancer in France are treated by investigational drugs or drugs available through pre-marketing compassionate use programmes. Such drugs were not considered in the present analysis.

#### Sociodemographic variables

The municipality of residence (commune) for each patient at the time of the initial hospitalisation was determined from their postcode. Patients whose postcode was not documented in the PMSI database were excluded from the analysis. The commune is the lowest tier of local authority in France and generally consists of a single population centre together with any surrounding hamlets or countryside, with a typical area of 10–50 km^2^. There are around 36,000 such municipalities in France, and these are grouped into 6000 geographical units which are coded in the PMSI database. Data were retrieved from the French national statistics office (INSEE) on the sociodemographic make-up of each municipality and used to classify them in terms of population density and social deprivation. Based on the national census data of 2011 and the surface area of the commune, the population density of the municipality (or group of municipalities) was categorised by quartile into four classes: very low (≤86 inhabitants/km^2^), low (87–309 inhabitants/km^2^), high (310–2073 inhabitants/km^2^) and very high (> 2073 inhabitants/km^2^) population density. Municipalities were ranked on the basis of a social deprivation index (SDI) determined on the basis of unemployment rate, median household income, the percentage of high school graduates in the adult population and the percentage of blue-collar workers in the active population [13]. This index has been validated previously in the French setting as a tool for evaluating socioeconomic disparities in health at the municipality level. All municipalities in France were divided by quartile into four classes, corresponding to most deprived, deprived, privileged and most privileged [13]. Population density and SDI are not correlated (Spearman’s rank correlation coefficient: *ρ* = 0.37).

### Statistical analysis

Logistic regression analyses were performed to calculate the odds ratios associated with patients prescribed FICHCOMP drugs. All models were multi-level, controlling for the population structure of the sample in order to take into account potential dependence between patients at the municipality level. In a first step, associations between the proportion of patients with access to FICHCOMP drugs and study variables of interest (age class, gender, presence of comorbidities, population density and SDI of the municipality of residence) were evaluated in univariate analyses using Fisher’s test. Only those variables showing significant (*p* < 0.10) associations with FICHCOMP drug use were entered into the multivariate analysis, which was performed using stepwise selection with backward elimination at a threshold of 0.05. Associations observed in the final model were expressed as odds ratios (OR) with their 95% confidence intervals (95% CI). Likelihood ratio testing was used for all tests of significance.

Statistical Analysis System software, version 9.2 for Windows (SAS Institute Inc., Cary, NC, USA) was used for all analyses.

## Results

### Study population

**Fig. 1:**
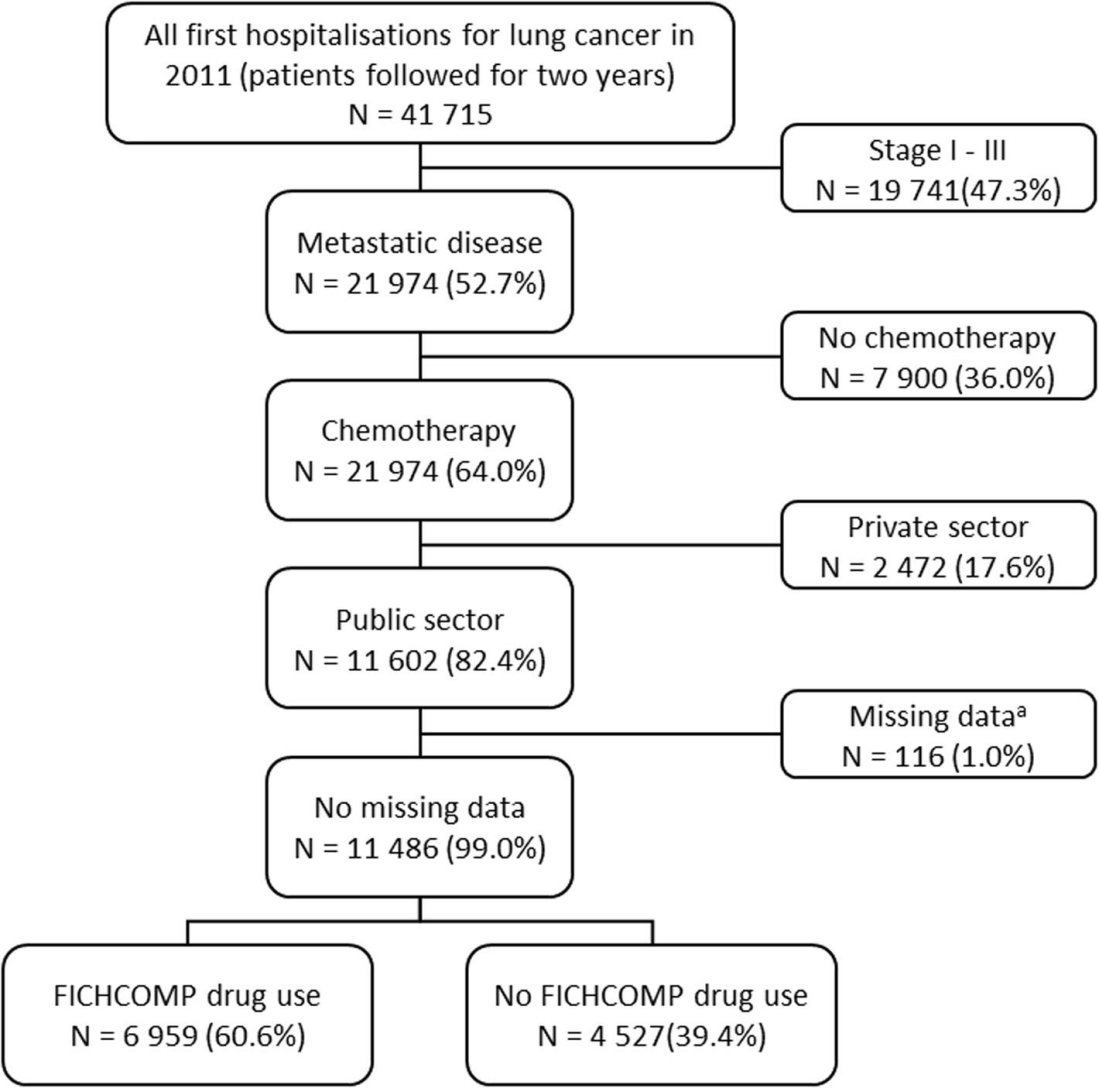
Patient disposition. ^a^For these 116 patients, the postcode of residence was unknown or missing

A total of 41,715 incident cases of lung cancer (all forms and all stages) were identified in the hospital discharge database in 2011, all of whom were followed for 2 years. Of these, 21,974 fulfilled the criteria for metastatic disease. These patients made 298,652 hospital visits, including both overnight stays and day hospitalisations for chemotherapy sessions, corresponding to an average of 13.6 visits per patient over the course of the follow-up period. Out of all incident patients, 64.0% received chemotherapy over the observation period, the majority in the public sector (82.4%). Of the 11,486 analysable patients receiving chemotherapy in the public sector, 6959 (60.6%) were treated at least once with a specific lung cancer drug (FICHCOMP). The flow chart for the selection of patients is presented in Fig. 1.

The characteristics of these patients are presented in Table 1. The mean age of all included patients at inclusion was 61.0 ± 10.1 years. The most frequent comorbidities were pulmonary diseases (notably COPD (chronic obstructive pulmonary disease)), hypertension and diabetes. Renal insufficiency was documented in 2.0% of patients. Around half of patients were managed in local community hospitals for their first stay.

**Table 1:** Characteristics of patients with metastatic lung cancer and with management of chemotherapy in the public sector and access to FICHCOMP drugs: univariate and multivariate logistic regression analysis (multi-level analysis)

	All patients (*N* = 11,486)		Patients with FICHCOMP drug use (*N* = 6959)		Univariate analysis	Multivariate analysis
	n	(% col)^a^	n	(% row)^b^	OR [95% CI]	OR [95% CI]
Age at inclusion (years)						
≤ 55 yrs	2923	(25.4%)	2058	(70.4%)	1.00	1.00
56–65 yrs	4206	(36.6%)	2659	(63.2%)	0.72 [0.65–0.80]	0.76 [0.69–0.84]
≥ 66 yrs	4357	(37.9%)	2242	(51.5%)	0.44 [0.40–0.49]	0.49 [0.44–0.55]
Gender						
Men	8172	(71.1%)	4819	(59.0%)	0.79 [0.72–0.86]	0.86 [0.79–0.94]
Women	3314	(28.9%)	2140	(64.6%)	1.00	1.00
Comorbidities						
Hypertension	2584	(22.5%)	1370	(53.0%)	0.68 [0.62–0.74]	0.88 [0.80–0.97]
Diabetes	1151	(10.0%)	567	(49.3%)	0.61 [0.54–0.69]	0.79 [0.69–0.90]
Renal insufficiency	224	(2.0%)	87	(38.8%)	0.41 [0.31–0.54]	0.55 [0.41–0.73]
COPD	1506	(13.1%)	744	(49.4%)	0.60 [0.54–0.67]	0.71 [0.63–0.80]
Pulmonary insufficiency	461	(4.0%)	225	(48.8%)	0.60 [0.50–0.73]	0.73 [0.60–0.88]
Other chronic lung diseases	855	(7.4%)	444	(51.9%)	0.68 [0.59–0.78]	0.93 [0.79–1.08]
Type of hospital for first stay with chemotherapy^c^						
CH	5615	(48.9%)	3405	(60.6%)	1.00	–
CHU	3284	(28.6%)	2019	(61.5%)	1.03 [0.95–1.13]	–
CLCC	1193	(10.4%)	716	(60.0%)	0.93 [0.81–1.06]	–
Others	1394	(12.1%)	819	(58.8%)	0.91 [0.80–1.03]	–
Population density of municipality (quartile; population nb hab/km^2^)^c^						
Very low density (≤ 86)	2891	(25.2%)	1693	(58.6%)	0.94 [0.83–1.06]	–
Low density (]86–309])	2905	(25.3%)	1743	(60.0%)	1.02 [0.91–1.15]	–
High density (]309–2073])	2872	(25.0%)	1772	(61.7%)	1.07 [0.95–1.21]	–
Very high density (> 2073)	2818	(24.5%)	1751	(62.1%)	1.00	–
Social deprivation of municipality (quartile)						
Most deprived	3031	(26.4%)	1762	(58.1%)	0.82 [0.73–0.93]	0.82 [0.72–0.92]
Deprived	3249	(28.3%)	1958	(60.3%)	0.90 [0.80–1.01]	0.87 [0.78–0.98]
Privileged	2461	(21.4%)	1493	(60.7%)	0.93 [0.82–1.04]	0.90 [0.80–1.02]
Most privileged	2745	(23.9%)	1746	(63.6%)	1.00	1.00

*COPD* chronic obstructive respiratory disease, *CH* community hospital, *CHU* university hospital, *CLCC* cancer care clinic, *OR* odds ratio, *95%CI* 95% confidence interval

^a^Percentage calculated with total number of patients as the denominator (11486). ^b^Percentage calculated as number of patients in class receiving FICHCOMP drugs. ^**c**^Non-significant variable in the univariate analysis (threshold = 10%), therefore not included in the multivariate analysis

### FICHCOMP drug use

The most widely prescribed FICHCOMP drug was pemetrexed, which was prescribed to 78.5% of patients receiving these drugs. Other FICHCOMP drugs prescribed to over 10 % of patients were bevacizumab and docetaxel (Table 2), although it should be noted that the latter was withdrawn from the special funding list during the data collection period.

**Table 2:** FICHCOMP anticancer drugs prescribed during the study

Any FICHCOMP drug	Patients	%	N° of stays	%
	*N* = 6959	100%	*N* = 42,220	100%
Pemetrexed	5464	78,5%	29,149	69.0%
Bevacizumab	1124	16,2%	8223	19.5%
Docetaxel^a^	1605	23,1%	5656	13.4%
Topotecan	350	5,0%	2245	5.3%
Gemcitabine^a^	280	4,0%	1035	2.5%

Patients could be prescribed more than one extra-DRG drug, so these frequency counts are not mutually exclusive

^a^These drugs were withdrawn from the special funding list (*liste en sus*) during the data collection period

### Variables associated with FICHCOMP drug use

In univariate analysis, patients prescribed FICHCOMP drugs were younger, more frequently women, less frequently presenting comorbidities and more frequently living in the most socially privileged municipalities (Table 1) than patients receiving other chemotherapy. No association was observed between access to FICHCOMP drugs and the type of hospital in which the patient was originally hospitalised (*p* = 0.19) or with the population density of the municipality in which the patient lived (*p* = 0.12).

In multivariate analyses (Table 1), the likelihood of prescription of FICHCOMP drugs was inversely related to age, being lower in patients ≥66 years compared to those ≤55 years (OR: 0.49 [0.44–0.55]). The likelihood of prescription was lower in men than in women (OR: 0.86 [0.79–0.94]). For all the comorbidities documented, with the exception of ‘other chronic lung diseases’, the presence of a comorbidity was associated with a lower likelihood of prescription of FICHCOMP drugs. This association was strongest for patients with renal insufficiency (OR: 0.55 [0.41–0.73]). With respect to sociodemographic variables, the association between FICHCOMP drug use and the social deprivation index remained significant. The likelihood of FICHCOMP drug prescription was significantly lower outside the most privileged municipalities, being lowest in the most deprived municipalities (OR: 0.82 [0.72–0.92]).

## Discussion

In this nationwide cohort study, potential differences in access to expensive anticancer medications funded through the French *liste en sus * system, which allows direct payment of the drug to the hospital on top of the DRG tariff, were evaluated. A significant reduction in access to medication as a function of social deprivation was observed. The proportion of patients prescribed a FICHCOMP drug ranged from 58.1% for patients living in the most socially deprived municipalities to 63.6% in the most socially privileged. The gradient in medication use across the SDI groups would argue against a spurious correlation. On the other hand, in the univariate analysis, no association of prescription of FICHCOMP medications with the population density of the municipality or with the type of hospital in which the patient was treated was observed. Associations were also observed between the likelihood of FICHCOMP drug prescription and age, gender and the presence of comorbidities. The lower likelihood of prescription in older patients and in patients with those comorbidities taken into account in treatment decisions (MCT meetings) may be explained by reticence to use these treatments in patients who are already frail. For some of these medications, the prescribing indication lists certain comorbidities and the elderly in the precautions for use or contra-indications of the medication. The reason why prescription rates are lower in men than in women is unclear. We have previously shown using the PMSI database that survival is better in women with incident metastatic lung cancer than in men [14], which may indicate that their treatment or disease trajectories are different to those of men.

This study was performed using data from the PMSI database, which is an exhaustive data on all patients hospitalised in France. Since diagnosis and management of cancer patients is exclusively hospital-based in France, it should be possible to identify all incident cases of lung cancer in this database at a national level. Indeed, the number of such cases of that we identified (41715) is close to the number of incident cases in France for 2012 documented by the National Cancer Institute (~ 40,000) [15]. The contemporary quality of coding in the PMSI database is considered to be high and a recent comparison of standardised incidence ratios for different types of cancer determined from the PMSI and from local cancer registries has shown that the two sources provide very similar estimates [16].

Inequalities in access to anticancer medications have been reported previously for several other countries including the United Kingdom [17], Australia [18], Canada [19], and the USA [20], but we are not aware of specific data relating to this issue from France. Two earlier studies of access to chemotherapy for lung cancer in England, one performed in the wealthy South-East [21] and the other in relatively poorer Yorkshire [22], reported differences in access as a function of social deprivation of a similar magnitude to our own study. In the former study [21], these differences were smaller than differences associated with age (higher prescription rates in younger patients) and notably with the cancer network responsible for the area in which the patient lived [23]. In our study, the health service catchment area appeared to be a less important determinant of access to medication. The North American studies have suggested that patients living in rural areas or far from hospitals have a lower access to treatment [24–26], a difference which was not observed in our study or in the British studies [17]. This difference probably reflects the much lower population density, and in consequence density of hospitals, in rural areas in North America compared to Europe. The absence of influence of rurality in access to innovative drug is also an important finding.

The present study cannot address whether the differences in access to FICHCOMP medications as a function of social deprivation has a relevant clinical impact. Nonetheless, in a previous analysis of the PMSI database [14], it was found that social deprivation was also associated with reduced survival following diagnosis both at the metastatic and non-metastatic stages. Likewise, the reason why patients living in socially deprived areas are less likely to be prescribed FICHCOMP drugs is also unclear. This is perhaps not due to a lower level of access to cancer care in general, since a recent observational study comparing socially vulnerable patients with lung cancer to less vulnerable patients found that the socially vulnerable declared consulting a general practitioner or an oncologist more often than non-vulnerable individuals [27].

This study also demonstrated that diffusion of innovative anticancer drugs in France was extensive, with around two-thirds of patients with metastatic disease in public hospitals being prescribed an anticancer treatment covered by extra-DRG funding. Prescription volumes of these drugs in France are several-fold higher than they are in the United Kingdom where a specific funding programme for expensive innovative anticancer drugs was introduced in 2010 [28]. This fund was aimed at addressing both the issue of restrictive reimbursement recommendations from the National Institute for Health and Care Excellence (NICE) and to correct for postcode prescribing. In France, before retrospective payment for expensive drugs on the expensive list was implemented, access to such drugs in the public sector was at the discretion of hospitals, which could choose whether or not to fund treatment on a case-by-case basis depending on their financial situation. The special funding mechanism for innovative drugs shelters physicians from the economic impact of their clinical choices on condition that they comply with a “good practice contract” signed with the regional health authorities. However, removing responsibility for the costs of care from the physician shifts that responsibility to the insurer (through drug coverage and management practices) and to the patient (through out-of-pocket cost sharing) [29].

The strengths of the present study include the population-based approach, with a cohort of all lung cancer patients managed in France in 1 year with two-years of follow-up. Nonetheless, the use of this data source presents certain drawbacks. Firstly, at the patient level, demographic variables were limited to gender and age. No information is available in the PMSI database on smoking status, tumour histology and staging, or functional performance. Histology is a major criterion for systemic treatment strategy and several FICHCOMP drugs are actually specific to non-squamous NSCLC. This is the case for the two most-widely used drugs in our study (pemetrexed and bevasizumab). The observed association between access to these drugs and social deprivation may thus be even larger if only non-squamous NSCLC had been considered. Secondly, socioeconomic variables could only be estimated at the level of the municipality of residence of the patient, which is only a proxy marker of individual socioeconomic status. Thirdly, it is also important to note that private hospitals, which account for one-fifth of lung cancer diagnoses in France did not enter data on the use of expensive drugs to the FICHCOMP database at the time of the study. For this reason, it is possible that the extent of use of these drugs and the determinants of use, may differ between the private and public sectors. Finally, it was also not possible to extent the analysis to access to oral tyrosine kinase inhibitors, since these are delivered in community medicine and are thus not available in the FICHCOMP database. At the time of this analysis only medications targeting EGFR were available. Molecular testing is carried out systematically on all patients with lung cancer in France [30] and EGFR mutations and ALK rearrangements are observed in around 16% of patients [31]. The proportion of patients with metastatic lung cancer who are eligible for treatment by tyrosine kinase inhibitors targeting EGFR is relatively low.

It should also be noted that treatment paradigms for metastatic lung cancer are rapidly evolving with the introduction of immunotherapies, the first of which was licensed in France in February 2016, now included in the FICHCOMP list. This development may have consequences for the pattern of use and access to FICHCOMP drugs for the treatment of metastatic lung cancer. The data collected in the present study will serve as a useful reference point to assess such changes in future studies.

## Conclusion

Access to innovative medication in France seems to be relatively equitable and the large disparities reported in other countries were not observed. In this sense, the introduction of the extra-DRG list of drugs (the *liste en sus*) can be considered to be relatively successful in ensuring rapid diffusion of innovative treatments to patients with lung cancer and in minimising geographical differences in access to treatment. Nevertheless, social deprivation appears to be associated with poorer access to medication, for reasons which needs to be identified. It is important to understand what underlies this association in order to propose and implement strategies to ensure equal access to anticancer treatments for people living in socially deprived areas. The issue of equity of access to innovative medicine will become increasingly important as new treatment options for lung cancer, and notably immunotherapies, become available.

## Abbreviations

CEPS: Comité Economique des Produits de Santé
CH: Community Hospital
CHU: Community University Hospital
CI: Confidence Interval
CLCC: Cancer Care Clinic
CNIL: Commission Nationale de l’Informatique et des Libertés
COPD: Chronic Obstructive Pulmonary Disease
DRG: Disease-Related Group
GPP: Good Pharmacoepidemiology Practices
ISPE: International Society for Pharmacoepidemiology
MCT: Multidisciplinary Care Team
NICE: National Institute for Health and Care Excellence
OR: Odds Ratios
PD: Principal Diagnoses
PMSI: Programme de Médicalisation des Systèmes d’Information
RD: Related Diagnoses
SAD: Significantly-Associated Diagnoses
SDI: Deprivation Index
